# Correction: Native architecture of the *Chlamydomonas* chloroplast revealed by in situ cryo-electron tomography

**DOI:** 10.7554/eLife.11383

**Published:** 2015-09-14

**Authors:** Benjamin D Engel, Miroslava Schaffer, Luis Kuhn Cuellar, Elizabeth Villa, Jürgen M Plitzko, Wolfgang Baumeister

Engel BD, Schaffer M, Cuellar LK, Villa E, Plitzko JM, Baumeister W. 2015. Native architecture of the *Chlamydomonas* chloroplast revealed by in situ cryo-electron tomography. *eLife* **4**:e04889. doi: 10.7554/eLife.04889

Published 13 January 2015

In the published article, the legend for Figure 3 stated ‘Scale bars: 200 μm’. This should have read ‘Scale bars: 200 nm’.

This has now been corrected.

